# Dyslipidemia and Inflammation as Hallmarks of Oxidative Stress in COVID-19: A Follow-Up Study

**DOI:** 10.3390/ijms232315350

**Published:** 2022-12-05

**Authors:** Álvaro Aparisi, Marta Martín-Fernández, Cristina Ybarra-Falcón, José Francisco Gil, Manuel Carrasco-Moraleja, Pedro Martínez-Paz, Iván Cusácovich, Hugo Gonzalo-Benito, Raúl Fuertes, Marta Marcos-Mangas, Carolina Iglesias-Echeverría, J. Alberto San Román, Eduardo Tamayo, David Andaluz-Ojeda, Álvaro Tamayo-Velasco

**Affiliations:** 1 Unidad de Cardiología Intervencionista, Servicio de Cardiología, Hospital del Mar, 08003 Barcelona, Spain; 2 Instituto Hospital del Mar de Investigaciones Médicas-IMIM, 08003 Barcelona, Spain; 3 Departamento de Medicina, Toxicología y Dermatología, Facultad de Medicina, Universidad de Valladolid, 47002 Valladolid, Spain; 4 Centro de Investigación Biomédica en Red de Enfermedades Infecciosas (CIBERINFEC), Instituto de Salud Carlos III, 28029 Madrid, Spain; 5 BioCritic, Group for Biomedical Research in Critical Care Medicine, 47005 Valladolid, Spain; 6 Servicio de Cardiología, Hospital Clínico Universitario de Valladolid, 47003 Valladolid, Spain; 7 Departamento de Cirugía, Facultad de Medicina, Universidad de Valladolid, 47005 Valladolid, Spain; 8 Servicio de Medicina Interna, Hospital Clínico Universitario de Valladolid, 47003 Valladolid, Spain; 9 Instituto de Ciencias de la Salud de Castilla y León (IECSCYL), 42002 Soria, Spain; 10 Servicio de Radiología, Hospital Universitario la Princesa, 28006 Madrid, Spain; 11 CIBER de Enfermedades Cardiovasculares (CIBERCV), 28029 Madrid, Spain; 12 Servicio de Anestesiología y Reanimación, Hospital Clínico Universitario de Valladolid, 47003 Valladolid, Spain; 13 Servicio de Medicina Intensiva, Hospital Universitario HM Sanchinarro, Hospitales Madrid, 28050 Madrid, Spain; 14 Servicio de Hematología y Hemoterapia, Hospital Clínico Universitario de Valladolid, 47003 Valladolid, Spain

**Keywords:** total cholesterol, lipoproteins, lipid peroxidation, oxidative stress, COVID-19, inflammation

## Abstract

Recent works have demonstrated a significant reduction in cholesterol levels and increased oxidative stress in patients with coronavirus disease 2019 (COVID-19). The cause of this alteration is not well known. This study aimed to comprehensively evaluate their possible association during the evolution of COVID-19. This is an observational prospective study. The primary endpoint was to analyze the association between lipid peroxidation, lipid, and inflammatory profiles in COVID-19 patients. A multivariate regression analysis was employed. The secondary endpoint included the long-term follow-up of lipid profiles. COVID-19 patients presented significantly lower values in their lipid profile (total, low, and high-density lipoprotein cholesterol) with greater oxidative stress and inflammatory response compared to the healthy controls. Lipid peroxidation was the unique oxidative parameter with a significant association with the total cholesterol (OR: 0.982; 95% CI: 0.969–0.996; *p* = 0.012), IL1-RA (OR: 0.999; 95% CI: 0.998–0.999; *p* = 0.021) IL-6 (OR: 1.062; 95% CI: 1.017–1.110; *p* = 0.007), IL-7 (OR: 0.653; 95% CI: 0.433–0.986; *p* = 0.042) and IL-17 (OR: 1.098; 95% CI: 1.010–1.193; *p* = 0.028). Lipid abnormalities recovered after the initial insult during long-term follow-up (IQR 514 days); however, those with high LPO levels at hospital admission had, during long-term follow-up, an atherogenic lipid profile. Our study suggests that oxidative stress in COVID-19 is associated with derangements of the lipid profile and inflammation. Survivors experienced a recovery in their lipid profiles during long-term follow-up, but those with stronger oxidative responses had an atherogenic lipid profile.

## 1. Introduction

Traditional risk factors have been suggested to be associated with higher mortality in Coronavirus disease 2019 (COVID-19) [1]. These conditions exhibit increased oxidative stress that leads to a series of downstream events impairing multiple biological processes and contributing to disease progression [2,3,4]. Increasing evidence suggests that oxidative stress may contribute to COVID-19 severity [5,6,7,8,9] and ultimately to its mortality [10]. Moreover, the literature also reveals that hospitalized COVID-19 patients with low plasma cholesterol [11,12] or previous dyslipidemia [13] levels are also at a higher risk for more severe disease and all-cause mortality [11,12].

Lipids are targets for oxidation, and through lipid peroxidation (LPO), their biological properties are modified through non-enzymatic or enzymatic chemical reactions [13]. Notably, phospholipids are a major target of oxidative damage, and their oxidation results in oxylipins that can regulate inflammation [14,15]. Lipoproteins consist of a phospholipid monolayer and apolipoproteins with a central core made up of lipophilic cholesterol esters and triglycerides. Under normal conditions, high-density cholesterol (HDL-c) and low-density cholesterol (LDL-c) have opposite biological functions [16]. During the acute-phase response of infection and inflammation, there is a reduction in the lipoprotein’s concentration [17]. In addition, a hypolipoprotein phenotype has been linked to diminished antioxidant capacity and the worst clinical outcomes in sepsis [18]. Interestingly, recent evidence has suggested the presence of oxidized LDL-c through the detection of autoantibodies for oxidized LDL-c in COVID-19 patients [19].

Based on this background, we aimed to evaluate a possible association between oxidative cell damage and lipid levels in COVID-19. Additionally, given the scarce knowledge about long-term metabolic alterations in COVID-19 survivors, we also evaluated the lipid profiles during long-term follow-up.

## 2. Results

The baseline characteristics and medication status of the 108 COVID-19 patients are detailed in Supplementary Table S1. When compared to the controls (*n* = 28), the median age (68.5 vs. 70.5 years; *p =* 0.296) and female gender (60.7% vs. 42.6%; *p* = 0.087) of the global COVID-19 cohort did not show any difference. Similarly, the prevalence of hypertension (46.3% vs. 50%), diabetes (17.6% vs. 14.3%), obesity (9.3% vs. 10.7%), and other comorbidities did not show any difference (*p* > 0.05).

### 2.1. The Inflammatory, Redox, and Lipid Profile of COVID-19 vs. Controls

We included a non-COVID-19 control group admitted for elective surgery to assess whether COVID-19 explained the aforementioned evaluated parameters (Table 1). Compared with the controls, COVID-19 cases were more likely to have significantly higher C-reactive protein (83.5 vs. 10 mg/dL), d-dimer (800 vs. 255 ng/mL), and lower lymphocyte count (945 vs. 2255 cells/mm^3^). In addition, plasma cytokines, total antioxidant capacity (FRAPS and ABTS), LPO, and protein carbonyls were significantly higher in COVID-19 patients as opposed to the controls. On the contrary, at the time of admission, the oxidative damage to DNA was comparable between the groups.

**Figure 1:**
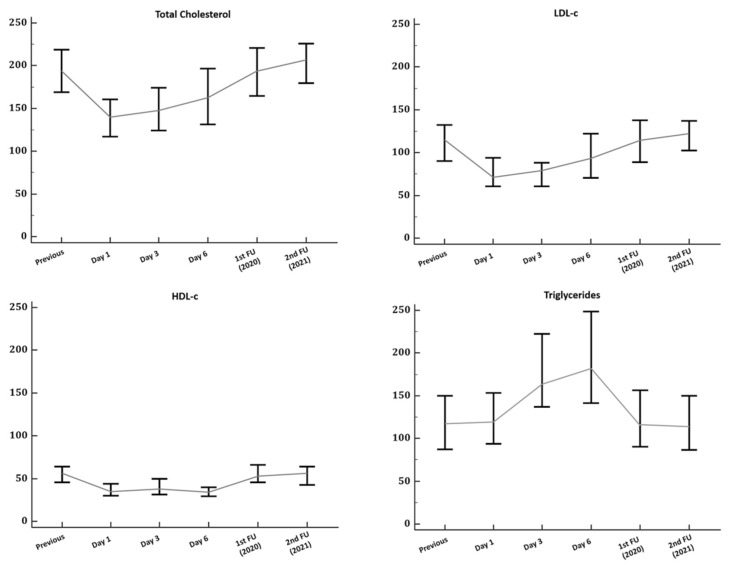
Temporal changes in lipid profiles in patients with a definitive diagnosis of COVID-19. Data in graphs are presented as median and the interquartile range with error bars representing 95% confidence intervals. Control patients were not included in this analysis. Abbreviations: FU—follow-up; HDL-c—high-density lipoprotein cholesterol; LDL-c—low-density lipoprotein cholesterol; TC—total cholesterol.

Patients with COVID-19 showed lower cholesterol and lipoprotein levels at the time of hospital admission compared to their previous basal levels (Figure 1). Compared to the controls at the time of hospital admission (Table 1), we observed significantly lower TC in patients with COVID-19 (140 vs. 175 mg/dL; *p* < 0.001), LDL-c (71.3 vs. 98 mg/dL; *p* = 0.002) and HDL-c (35 vs. 59 mg/dL; *p* < 0.001), but also greater TG (119 vs. 89.5 mg/dL; *p* = 0.027) levels.

### 2.2. Association of Lipid Peroxidation with Lipid and Inflammatory Markers

To explore the possible interactions between lipid, inflammation, and oxidative stress, a correlation analysis was performed (Supplementary Table S2). LPO showed a negative correlation with TC (*r* = −0.345, *p* < 0.001), HDL-c (*r* = −0.319, *p* = 0.001), and LDL-c (*r* = −0.213, *p* = 0.035) with the strongest correlation with LDL-c (*r* = −0.637, *p* = 0.019) in COVID-19 non-survivors. On the contrary, the correlation of LPO with the main cytokines showed a positive correlation with IL1RA (*r* = 0.314, *p* < 0.001), IL-1β (*r* = 0.192, *p* = 0.027), IL-6 (r = 0.242; *p = 0.005*), IL-17α (*r* = 0.288, *p* < 0.001), IL-18 (*r* = 0.329, *p* < 0.001), and IP-10 (*r* = 0.538, *p* < 0.001).

A logistic regression model was used to examine the association between LPO with lipid and inflammatory profiles (see Table 2). A cut-off value of LPO > 1948.17 μM was chosen as it was assumed to be associated with greater mortality [10]. Variables independently associated with the LPO > 1948.17 μM were total cholesterol (OR: 0.982; 95% CI: 0.969–0.996; *p* = 0.012), IL1-RA (OR: 0.999; 95% CI: 0.998–0.999; *p* = 0.021) IL-6 (OR: 1.062; 95% CI: 1.017–1.110; *p* = 0.007), IL-7 (OR: 0.653; 95% CI: 0.433–0.986; *p* = 0.042), and IL-17 (OR: 1.098; 95% CI: 1.010–1.193; *p* = 0.028). We did not find a significant association between LPO with age or sex. Of interest, the final model did not include lymphocyte count as it was strongly correlated with the total cholesterol (r = 0.394; *p* < 0.001), suggesting potential collinearity.

### 2.3. Follow-Up

Among the COVID-19 survivors, there was a significant recovery (*p* < 0.001) of TC, HDL-c, and LDL-c from hospital admission to the first-time follow-up (IQR 79 [68–93] days). The recovery persisted during the second follow-up (IQR 514 [427–617] days) but did not result in significant increases compared with the previous follow-up (Supplemental Table S3). Compared with the controls, we found that only TG levels were significantly higher in post-COVID-19 patients during the first (91 vs. 115 mg/dL; *p* = 0.02) and second follow-up (84 vs. 112 mg/dL; *p* = 0.003).

To evaluate whether oxidate stress at admission was related to long-term lipid abnormalities, subjects were stratified into two groups according to our previous estimated cut-off values for LPO [10]. Subsequently, no significant differences were found in lipid or inflammatory markers during the first follow-up. However, we observed during the second follow-up higher TC (207 vs. 192 mg/dL; *p* = 0.026), LDL-c (131 vs. 114 mg/dL; *p* = 0.025), TG (127 vs. 109 mg/dL; *p* = 0.002) and TC/HDL-c (3.4 vs. 3.9; *p* = 0.003), but with low HDL-c (48 vs. 57; *p* = 0.031) levels among patients with a higher oxidative response (LPO > 1948 μM) at the time of admission (Table 3).

### 2.4. Clinical Outcomes and Mortality

Regarding clinical outcomes, the 90-day all-cause mortality rate was 22.2% in the global COVID-19 cohort. Figure S1 shows unadjusted Kaplan–Meier curves of 90-day all-cause mortality between the groups according to the previous estimated cut-off values for LPO [10] and LDL [20]. Both LPO > 1948 μM and LDL < 69 mg/dL were significantly associated with higher mortality. During a median of 514 (427–617) days of follow-up, no deaths were reported.

## 3. Discussion

This prospective study investigated the association between the lipid profile, oxidative stress, and inflammatory markers of patients with COVID-19 compared to the healthy controls. Several findings have emerged: (1) COVID-19 patients had significantly lower cholesterol levels with higher plasma cytokine and oxidative stress levels compared to the control patients at the time of hospital admission; (2) high LPO levels were independently associated with TC and proinflammatory cytokines (IL-1RA, IL-17, and IL-7); (3) lipid profiles rapidly recovered among survivors after hospital discharge, but those with previous high LPO at hospital admission showed an atherogenic lipid profile during long-term follow-up.

The evolution of sequential changes in cholesterol levels and lipoproteins over the hospital admission and follow-up period suggested COVID-19-mediated dyslipidemia. Compared to the healthy controls, we observed that the inflammatory and oxidative profiles of COVID-19 patients were significantly higher at the index examination, whereas their lipid profile was significantly lower. This laboratory phenotypic profile raised the possibility as to whether inflammation and oxidative stress are important drivers of observed dyslipidemia. To that end, we found that elevated LPO levels were independently associated with low cholesterol levels (OR: 0.982; 95% CI: 0.969–0.996), low IL1RA (OR: 0.999; 95% CI: 0.998–0.999), and low IL-7 (OR: 0.653; 95% CI: 0.433–0.986), but with higher IL-6 (OR: 1.062; 95% CI: 1.017–1.110) and IL-17A (OR: 1.098; 95% CI: 1.010–1.193) levels. These observations, combined with the independent association of LPO [10], lipoproteins [11,12], and cytokines [21] with increased mortality with COVID-19, support a possible link.

Some hypotheses arise from our findings concerning the mechanism(s) behind the observed association(s) [22,23]. First, high LPO with low IL1RA and high IL-6 levels could be attributed to inflammasome activation [24]. This is in line with the evidence reported by SL Lage et al., who observed a strong correlation between LPO and activation of pyrin domain-containing 3 (NLRP3)-inflammasome in patients with COVID-19 [25]. Similarly, the association between high LPO and low cholesterol levels could be explained by the oxidative modification of lipoproteins [26,27], which, in turn, can also activate the inflammasome and its downstream mediators [28,29]. However, some discrepancies in disease severity and mortality have been observed with lipoproteins [11,12] as opposed to oxidative stress. Although these observations are likely the result of a combination of many factors, we believe that such discrepancies are the result of the initial acute phase response. In this sense, one would expect low initial levels of HDL-c [22,30], given that it is more prone to oxidation until its antioxidant capacity is overwhelmed, leading to LDL-c oxidation at later stages. This concept is supported by recent studies showing that severe COVID-19 patients had a pro-inflammatory oxylipin profile [6], and a higher degree of LDL-c oxidation is associated with a greater concentration of oxylipins [31]. Whether HDL-c and LDL-c could serve as a biomarker of disease progression and their association with LPO merits investigation for a better risk assessment.

Another intriguing observation was the association between LPO with IL-7 and IL-17A levels, which may indicate a mechanism by which COVID-19 leads to a cytokine storm [21] and lymphopenia [32]. IL-17 is produced by T-helper 17 cells and is considered a pro-inflammatory cytokine, which has been studied in autoimmune diseases, and infectious diseases and is associated with lipid metabolic disorders [33,34,35]. Therefore, the overexpression of IL-17 may lead to the increased production of reactive oxygen species [36], which can enhance the inflammatory response [33] and can be an important driver of the observed cytokine storm in some COVID-19 patients [21]. On the other hand, the decreased IL-7 levels may explain the associated lymphopenia in the most severe cases as a result of exhaustion, as it is required for T-helper 17 development [32,37]. Nevertheless, oxidative stress may lead to lymphopenia through the diminished responsiveness of T-cells to IL-7, as observed in the human immunodeficiency virus [38]. Overall, our findings suggest complex crosstalk between oxidative stress with lipid metabolism and inflammatory response likely creates a vicious circle that propagates the disease process.

Interestingly, data about the long-term effects of sepsis or COVID-19 among survivors on lipid profiles are scarce. A population-based matched cohort study of 54,241 sepsis survivors showed a higher rate of major cardiovascular events compared to non-sepsis survivors (HR 1.30; 95% CI 1.27–1.32) during 5 years of follow-up [39]. Similarly, data from a recent study found an association with major adverse cardiovascular events among COVID-19 survivors during 12-month follow-up in comparison to contemporary (HR 1.42; 95% CI 1.38–1.47) and historical (HR 1.47; 95% CI 1.43–1.52) control groups [40]. However, little is known about the potential physiologic changes behind long-term related cardiovascular outcomes in septic patients.

An unresolved inflammatory response or persistent inflammasome activation can promote vascular disease via self-supporting mechanisms [29], in which oxidative stress can lead to chronic metabolic disorders and impact the cardiovascular system [3]. Of note, one study observed among sepsis survivors increased levels of TC with a blunted response to statin therapy after 2 years of follow-up [41]. Conversely, a recent study reported that COVID-19 patients showed persistent oxidative stress and inflammasome activation [25]. Based on these assumptions, we hypothesized that patients with higher LPO at admission might have an atherogenic lipid profile during follow-up. According to our results, patients with higher LPO at admission showed an atherogenic lipid profile with a tendency toward a low-grade systemic inflammation during long-term follow-up. Similar findings were described in other infectious diseases when compared to the healthy controls after 4 months of follow-up, mostly characterized by low HDL-c levels and low paraoxonase-1 activity [42,43].

At present, it remains to be established how previous infections can increase the risk of cardiovascular events. Although this study does not report information about LPO during long-term follow-up, our previous findings might be explained because persistent LPO leaves HDL-c susceptible to oxidation and impairs paraoxonase-1 activity [27]. In addition, a low HDL-c phenotype has been associated before with a greater rate of LPO and platelet activation [44]. Whether COVID-19 will increase the risk of cardiovascular disease is not known, but we believe that this observation also requires careful assessment [29].

### Limitations

Our study has limitations inherent to observational studies despite its prospective design. First, we did not measure directly oxidized forms; however, MDA and 4-HNE concentrations were measured as an index of LPO and were found in ox-LDL [45]. Second, the association between lipid, oxidative, and inflammatory profiles was only measured at the time of the index examination to minimize the confounding effect of other variables. Finally, our study is the first to provide data on the long-term follow-up of lipid profiles in COVID-19 survivors, but we did not assess lifestyle or lipid-lowering treatment modifications that could result in lipid changes during this period. Despite its limitations, our findings may provide a rationale for future large-scale prospective studies to replicate our findings and understand the mechanism underlying the observed associations.

## 4. Materials and Methods

### 4.1. Study Design and Patient Inclusion Criteria

This is an observational prospective study of patients with COVID-19 from a single tertiary center in Spain (Hospital Clínico Universitario de Valladolid, Valladolid, Spain). Participants were recruited between the 24th of March and the 11th of April 2020 during the first wave. Survivors underwent follow-up until December 2021 after hospital discharge.

Eligible participants had a positive nasopharyngeal swab reverse transcriptase-polymerase chain reaction for SARS-CoV-2 infection. Exclusion criteria were (1) age < 18 years, (2) history of any severe chronic or terminal illness, (3) pregnancy, and (4) any other acute disease or infection at the time of admission. COVID-19 patients were compared with control healthy subjects, who were admitted during the same period for scheduled surgery in a ratio of 4:1 (cases/controls). The control subjects were asymptomatic and had negative nasopharyngeal swab reverse transcriptase-polymerase chain reaction for SARS-CoV-2 infection before the pre-anesthetic evaluation.

The study was reviewed and approved by the Institutional Review Ethics Committee of the H. Clínico de Valladolid (PI 20-1717). Each participant or their legal representatives provided written informed consent before recruitment in this prospective study. This study complied with the code of ethics for the World Medical Association (Declaration of Helsinki).

### 4.2. Biological Samples

Blood samples were collected after an overnight fast to avoid circadian variations. The samples were centrifuged at 2000× *g* for 20 min at room temperature, and the serum was stored at −80 °C until analysis. Measurements of the complete blood cell count and biomarkers of inflammatory status were measured as previously reported elsewhere [46]. All laboratory samples were blindly assessed, randomly distributed to avoid biases in the experimental procedures, and evaluated no later than three months after the inclusion period [46].

### 4.3. Lipid Profile Analysis

HDL-c, LDL-c, total cholesterol (TC), and triglycerides (TG) were tested on the Roche Cobas 8100 sampling system analyzer (Module Cobasc 702, Roche Diagnostics, Switzerland). Direct LDL-c, HDL-c, and TC measurements were assessed according to standard homogeneous enzymatic colorimetric assays in accordance with instructions from the manufacturer.

### 4.4. Cytokine Analysis

Plasma cytokine quantification was performed according to the manufacturer’s instructions with the commercially available 45-Plex Human ProcartaPlex™ Panel 1 (Invitrogen, Waltham, MA, USA) from store aliquots. All cytokines had at least 20% detection to ensure the robustness of the results and were expressed in logarithm base 2 [46]. The quantified interleukins (IL) included the IL-1 alpha, IL-1 beta, IL-1 receptor antagonist (IL-1RA), IL-2, IL-4, IL-5, IL-6, IL-7, IL-8/CXCL8, IL-9, IL-10, IL-12 p70, IL-13, IL-15, IL-17A, IL-18, IL-21, IL-22, IL-23, IL-27, and IL-31. Other evaluated cytokines are listed in Supplementary Materials.

### 4.5. Oxidant Cell Damage and Antioxidant Capacity Determination

One of the consequences of uncontrolled oxidative stress is oxidative damage to lipids, nucleic acids, and proteins. The evaluation of lipid peroxidation was part of the study protocol; we measured the plasma levels of malondialdehyde (MDA) and 4-hydroxynonenal (4-HNE). Both are LPO end-products that are generated in vivo by the degradation of polyunsaturated fatty acids and were estimated by the use of the Bio-quochem commercial kit ref KB03002 (BQCellTM MTT, Bioquochem, Oviedo, Spain) following the manufacturer’s recommendations. Reactions between the indoles and aldehydes (MDA and HNE) give a diindolylalkane (chromophore) whose maximal absorbance is in the 580–620 nm region.

To investigate systemic oxidative stress-induced DNA damage, plasma levels of 8-hydroxy-2′-deoxyguanosine (8-OHdG) were quantitatively measured at a 450 nm wavelength by using The DetectX^®^ DNA Damage Immunoassay Kit (Arbor Assays, Ann Arbor, MI, USA). We also evaluated protein carbonylation, a non-reversible covalent modification of proteins by LPO end-products, by using the Protein Carbonyl Colori-metric Assay Kit (Tissue and Serum Samples) commercial kit ref E-BC-K117-S (Elabscience Biotechnology Inc., Houston, TX, USA). Both were measured according to the manufacturer’s recommendations.

The cumulative action of the total antioxidant capacity was quantified by two methods: FRAP (ferric reducing antioxidant power) and ABTS (2,2-azino-bis (3-ethylbenzthioziozline-6-sulfonic acid). Both techniques have been described elsewhere and followed manufacturers’ recommendations [10].

### 4.6. Endpoints

The primary endpoint of the study was to evaluate the association between lipid peroxidation with lipid profile and inflammatory response. To determine this, we performed a cross-sectional analysis on the day of hospital admission. The secondary endpoint included the long-term effects of COVID-19 on the lipid profile of survivors.

### 4.7. Statistical Analysis

Categorical variables are reported as absolute values and percentages. Continuous variables were presented as the median and interquartile range (IQR). The normality of continuous variables was verified with the Kolmogorov–Smirnov test and Q–Q plot. Categorical variables were compared with the chi-square test and the Fisher exact test to assess the significance of differences between the groups. The comparison of continuous variables was performed with the Mann–Whitney U test or Wilcoxon signed-rank test for paired comparisons.

A Spearman’s correlation test was performed to analyze the correlation between oxidative stress, the lipids profile, and inflammatory markers. To identify factors that were predictive of high LPO levels, a logistic regression model with the maximum likelihood method was constructed by using forward stepwise selection, which included the variables that were statistically significant in the bivariate analysis. No more than 1 variable per 10 outcome events was entered into the logistic model to avoid overfitting. For the final model, we calculated the adjusted odds ratios (OR) for each of the included variables, along with their 95% confidence intervals (CIs). The goodness of fit for each model was determined with the Hosmer–Lemeshow test and area under the curve (AUC). Changes in TC and Lipoprotein concentrations during follow-up were compared according to LPO levels at admission with an analysis of covariance (ANCOVA); the model was adjusted for sex and time to follow-up. The unadjusted Kaplan–Meier risk estimates for the 90-day all-cause mortality were plotted for each assessed variable, and differences were evaluated with the Konp-chi test.

All statistical analyses were carried out by using the R software version 3.6.1 (R Project for Statistical Computing), and for the non-parametric test, KONP-based Cauchy-combination with the R-Package KONPsurv (v.1.0.4, Matan Schlesinger and Malka Gorfine). Differences were considered statistically significant when the *p*-value was < 0.05.

## 5. Conclusions

In summary, our findings suggest that predictors of lipid peroxidation in COVID-19 are cholesterol and interleukin levels. Future studies should focus on these associations during COVID-19 hospitalization and compare them between survivors and non-survivors. Moreover, the routine measurement of lipid profiles should be considered during long-term follow-up following COVID-19 hospitalization, although its benefit in terms of risk stratification and clinical decision-making in this setting is unknown.

## Figures and Tables

**Table 1:** Lipid, inflammatory, and oxidative stress profiles in COVID-19 patients vs. healthy controls at the time of hospital admission.

	Global COVID-19 (*n* = 108)	Controls (*n* = 28)	*p*-Value
**Inflammatory markers**			
**CRP, mg/L**	83.5 [38–151.2]	10 [7–10]	**<0.001**
**D-dimer, ng/mL**	800 [470–1713]	255 [224–330]	**<0.001**
**Ferritin, ng/mL**	731 [412–1500]	NA	NA
**Lymphocytes, cells/mm^3^**	945 [630–1221]	2255 [1545–2485]	**<0.001**
**Cytokines and chemokines**			
**IL-1β, pg/mL**	6.62 [2.71–13.2]	2.49 [1.76–2.81]	**<0.001**
**IL-1RA, pg/mL**	604.7 [258.6–1282.5]	72.5 [45.9–87.1]	**<0.001**
**IL-6, pg/mL**	13.07 [6.57–29.45]	8.69 [5.95–10.01]	**0.010**
**IL-7, pg/mL**	1.67 [0.72–3.58]	0.72 [0.50–0.82]	**<0.001**
**IL-17A, pg/mL**	7.03 [3.11–18.33]	2.03 [1.59–2.32]	**<0.001**
**IL-18, pg/mL**	47.2 [25–76.55]	14.53 [11.89–32.3]	**<0.001**
**IP-10, pg/mL**	45.55 [29.07–71.30]	5.56 [3.07–7.34]	**<0.001**
**Oxidative markers**			
**8-OHdG ^a^, pg/mL**	8373 [5445–12,497]	7925 [5130–9722]	0.246
**ABTS ^b^** **, µM**	2264 [1938–2462]	2510 [2371–2768]	**<0.001**
**FRAP ^b^** **, µM**	453.8 [385.7–576.1]	700.7 [554.1–796.4]	**<0.001**
**LPO ^c^** **, µM**	2123 [1250–3249]	284 [174–509]	**<0.001**
**Protein carbonyl ^d^** **, nmol/mg prot.**	10.8 [7.2–14.6]	5.6 [4.4–8.07]	**<0.001**
**Lipid profile**			
**Total cholesterol, mg/dL**	140 [117.5–159.5]	175 [164–197]	**<0.001**
**LDL-c, mg/dL**	71.3 [60.4–93.5]	98 [84.4–125.6]	**0.002**
**HDL-c, mg/dL**	35 [29.2–44]	59 [50.4–65.3]	**<0.001**
**TG, mg/dL**	119 [94.5–153.5]	89.5 [83–127]	**0.027**
**TC/HDL-c**	3.68 [3.18–4.66]	2.9 [2.52–3.83]	**0.008**

Values are expressed as median (IQR). Significant values (*p* < 0.05) are **bold.** Abbreviations: ABPS—2,2-azino-bis (3-ethylbenzthioziozline-6-sulfonic acid; CRP—C-reactive protein; FRAP—ferric reducing antioxidant power; HDL-c—high-density cholesterol; IL—interleukin; IP-10—Interferon-γ-Inducible Protein 10; LDL-c—low-density cholesterol; LPO—lipid peroxidation; NA—not applicable; TC/HDL-c—total cholesterol/high-density cholesterol ratio; TG—triglycerides. ^a^ Evaluates oxidative DNA damage, ^b^ Evaluates total antioxidant capacity, ^c^ Evaluates oxidative damage to lipids, ^d^ Evaluates oxidative damage to proteins.

**Table 2:** Univariable and multivariable logistic regression analysis of lipid peroxidation at the time of hospital admission.

Variable	Univariable		Multivariable	
	OR (95% CI)	*p*-Value	OR (95% CI)	*p*-Value
**Age**	1.007 (0.971–1.045)	0.700		
**Male gender**	0.883 (0.320–2.435)	0.810		
**Total cholesterol**	0.986 (0.969–1.002)	0.092	0.982 (0.969–0.996)	**0.012**
**IL1RA**	0.999 (0.998–0.999)	0.017	0.999 (0.998–0.999)	**0.021**
**IL-2**	0.961 (0.862–1.070)	0.467		
**IL-4**	0.991 (0.825–1.191)	0.927		
**IL-5**	0.996 (0.976–1.017)	0.703		
**IL-6**	1.094 (1.026–1.166)	0.006	1.062 (1.017–1.110)	**0.007**
**IL-7**	0.582 (0.311–1.087)	0.089	0.653 (0.433–0.986)	**0.042**
**IL-8**	0.983 (0.952–1.015)	0.302		
**IL-9**	0.797 (0.512–1.240)	0.314		
**IL-12p70**	0.856 (0.480–1.528)	0.600		
**IL-15**	1.018 (0.931–1.112)	0.702		
**IL-17α**	1.200 (0.998–1.442)	0.052	1.098 (1.010–1.193)	**0.028**
**IL-18**	1.010 (0.996–1.023)	0.164		
**IL-23**	1.449 (0.951–2.208)	0.084		
**IL-31**	0.735 (0.535–1.009)	0.057		
**IP-10**	0.995 (0.987–1.003)	0.225		

The outcome of interest is LPO > 1948.17 μM, which is independently associated with COVID-19 mortality [10]. Significant values (*p* < 0.05) are **bold**. Abbreviations: IL—interleukin; IP-10—interferon-γ-inducible protein 10; LPO—lipid peroxidation; OR—Odds ratio.

**Table 3:** Change in plasma lipid and lipoprotein concentrations between baseline and follow-up levels according to lipid peroxidation at admission.

	LPO ≤ 1948 μM ^a^	LPO > 1948 μM ^a^	ANCOVA Adjusted *p*-Value ^d^
**First follow-up ^b^**			
**TC, mg/dL**	186 [163–210]	195 [157–215]	0.763
**LDL-c, mg/dL**	123 [102–139]	107 [102–135]	0.475
**HDL-c, mg/dL**	58 [47–66]	49 [36–67]	0.392
**TG, mg/dL**	107 [83–139]	115 [101–168]	0.372
**TC/HDL-c**	3.4 [2.9–4.5]	3.5 [2.8–5.2]	0.306
**CRP, mg/L**	1.6 [1–4.3]	1.7 [1–4.3]	0.703
**Second follow-up** **^c^**			
**TC, mg/dL**	192 [174–211]	207 [197–230]	**0.026**
**LDL-c, mg/dL**	114 [99–128]	131 [120–147]	**0.025**
**HDL-c, mg/dL**	57 [45–65]	48 [38–62]	**0.031**
**TG, mg/dL**	109 [86–127]	127 [107–150]	**0.002**
**TC/HDL-c**	3.4 [2.9–3.6]	3.9 [3.4–4.8]	**0.003**
**CRP, mg/L**	2.5 [1–5.25]	5.8 [2–15]	0.072

Abbreviations: CRP—C-reactive protein; HDL-c—high-density cholesterol; LDL-c—low-density cholesterol; LPO—lipid peroxidation; TC—total cholesterol; TC/HDL-c—total cholesterol/high-density cholesterol ratio; TG—triglycerides. Values are reported as median (IQR). Significant values (*p* < 0.05) are bold. ^a^ Previously estimated through the optimal operating point of LPO as described in ref. [10]. ^b^ Median follow-up time of the global COVID-19 survivors’ cohort was 79 [68–93] days, no differences were observed between groups (*p* > 0.05). ^c^ Median follow-up time of the global COVID-19 survivors cohort was 514 [427–617] days, no differences were observed between groups (*p* > 0.05). ^d^ When the Levene test was significant, we used log_10_-transform. The normality of residuals was verified.

## Data Availability

The data presented in this study are available upon reasonable request from the corresponding author.

## Supplementary Materials

The following supporting information can be downloaded at: https://www.mdpi.com/article/10.3390/ijms232315350/s1.

## Abbreviations

COVID-19	Coronavirus disease 2019
CI	Confidence interval
CRP	C-reactive protein
IL	Interleukin
HDL-c	High-density lipoprotein cholesterol
LDL-c	Low-density lipoprotein cholesterol
LPO	Lipid peroxidation
NLRP3	Pyrin domain-containing 3
SARS-CoV-2	Severe acute respiratory syndrome coronavirus 2
TC	Total cholesterol
TG	Tryglycerides
