# Supplementary material for: Dyslipidemia and Inflammation as Hallmarks of Oxidative Stress in COVID-19: A Follow-Up Study
Source: Int J Mol Sci. 2022 Dec 5;23(23):15350. doi: 10.3390/ijms232315350 (PMC9736368; doi:10.3390/ijms232315350)
Supplement: Supplementary file 1 [file ijms-23-15350-s001.zip › ijms-1976589-supplementary.pdf]

SUPPLMENTARY MATERIALS

Supplemental material

Other evaluated cytokines were: brain-derived neurotrophic factor (BDNF), Eotaxin/CCL11, epidermal growth factor (EGF), fibroblast growth factor 2 (FGF-2), granulocyte macrophage colonystimulating factor (GM-CSF), growth-regulated oncogene (GRO) alpha/chemokine (C-X-C motif) ligand 1(CXCL1), hepatocyte growth factor (HGF), nerve growth factor (NGF) beta, leukaemia inhibitory factor (LIF), interferon (IFN) alpha, IFN gamma, interferon gamma-induced protein 10 (IP-10)/ chemokine (C-X-C motif) ligand 10 (CXCL10), monocyte chemoattractant protein 1 (MCP-1)/chemokine (C-C motif) ligand 2 (CCL2), macrophage inflammatory protein 1 (MIP-1) alpha/ (chemokine (C-C-motif) ligand 3(CCL3), regulated upon activation normal T Cell expressed and presumably secreted (RANTES)/ chemokine (C-C motif) ligand 5 (CCL5), stromal-cell derived factor 1 (SDF-1) alpha/CXCL12, tumoral necrosis factor (TNF) alpha, TNF beta/lymphotoxin alpha (LTA), platelet-derived growth factor (PDGF)- BB, placental growth factor (PIGF-1), stem cell factor (SCF), vascular endothelial growth factor (VEGF)-A and VEGF-D**.**

SUPPLEMENTARY TABLE

Table S1. Baseline characteristics of the global COVID-19 population

**Baseline characteristics**	**Global (n =108)**
**Age, years**	68.5 [59-75.5]
**Male**	62 (57.4)
**CAD**	10 (9.3)
**CKD**[a]	3 (2.8)
**Diabetes mellitus**	19 (17.6)
**Dyslipidaemia**[b]	36 (35.6)
**Hypertension**	50 (46.3)
**Obesity**[c]	10 (9.3)
**Prior Lung disease**[d]	19 (18.8)
**Neurological disease**[e]	3 (2.8)
**Chronic treatment**	
**Antiplatelets**	14 (13.9)
**Anticoagulants**	10 (9.9)
**Beta-blockers**	12 (11.2)
**RAAS inhibitors**	33 (31.1)
**Statin**	33 (32.7)

Values are expressed as median (interquartile range) or n (%).

Abbreviations: CAD: coronary artery disease; CKD = chronic kidney disease

[a] Defined as glomerular filtration rate <60 ml/min or need of intermittent renal replacement therapy

[b] Defined as chronic lipid lowering treatment (> 3 months)

[c] Defined as body mass index ≥ 30 kg/m^2

[d] Includes chronic obstructive pulmonary disease and other respiratory conditions

[e] Includes stroke and other neurological conditions

Table S2. Correlation analysis of lipid peroxidation, lipid profiles and cytokines at the time of admission

	Lymphocytes	CRP	Ferritin	IL-1β	IL1RA	IL-6	IL-18	IL-17α	IFNγ	IP10	LPO
**Total cholesterol**	0.393**	-0.242*	-0.032	-0.237*	-0.393**	-0.223*	-0.373**	-0.177	0.067	-0.429**	-0.345**
**HDL-c**	0.266**	-0.175	-0.053	-0.243*	-0.300**	-0.201*	-0.290*	-0.199*	-0.016	-0.412**	-0.319**
**LDL-c**	0.386**	-0.296**	-0.132	-0.161	-0.332**	-0.143	-0.306*	-0.094	-0.021	-0.376**	-0.213*
**Triglycerides**	-0.107	0.246*	0.331**	0.130	0.139	0.112	0.127	0.131	-0.142	0.127*	0.130
**Lymphocytes**	NA	0.017	-0.213*	-0.218*	-0.264*	0.079	-0.241*	-0.214*	0.135	-0.473**	-0.427**
**LPO**	-0.427**	0.005	0.233*	0.192*	0.314**	0.242**	0.329*	0.288**	0.028	0.538*	NA

Significant values * ($p < 0.05$); ** ($p < 0.001$) and were derived from Spearman correlation analysis

Abbreviations: CRP: C-reactive protein; FRAP: ferric reducing antioxidant power; HDL-c: high density cholesterol; IL: interleukin; IP-10: interferon-γ-inducible protein 10; LDL-c: low density cholesterol; LPO: lipid peroxidation; NA: not applicable

Table S3. Mean Change in Plasma Lipid and Lipoprotein concentrations between Baseline and Follow-up levels in COVID-19 survivors

	Baseline[a]	Follow-up	p-value [d]
**First follow-up [b]**			
**Total cholesterol, mg/dL**	143 [117-164]	197 [166-221]	**<0.001**
**LDL-c, mg/dL**	80.8 [54.5-95.9]	117.9 [88.1-138.9]	**<0.001**
**HDL-c, mg/dL**	35.6 [29.1-48.7]	51.9 [42.8-65.8]	**<0.001**
**TG, mg/dL**	120 [93-160]	115 [90-147]	0.418
**TC/HDL-c**	3.7 [3.2-4.8]	3.5 [2.9-4.9]	0.470
**Ferritin, µg/mL**	670 [406-1,164]	156 [78-266]	**<0.001**
**Lymphocytes, cells/mm^3**	1,000 [727-1,285]	1,985 [1,500-2,565]	**<0.001**
**CRP, mg/dL**	77 [36.5-130.5]	1.6 [1-3.7]	**<0.001**
**Second follow-up [c]**			
**Total cholesterol, mg/dL**	142 [117-162]	204 [185-228]	**<0.001**
**LDL-c, mg/dL**	80.5 [61-96]	124.6 [106.5-137]	**<0.001**
**HDL-c, mg/dL**	32.3 [29.1-43.7]	56 [42-64]	**<0.001**
**TG, mg/dL**	120 [97-154]	111 [87-150]	0.790
**TC/HDL-c**	3.8 [3.2-4.8]	3.6 [3.2-4.8]	0.504
**Ferritin, µg/mL**	640 [312-1,140]	102 [53-218]	**<0.001**
**Lymphocytes, cells/mm^3**	1,000 [732-1345]	2,195 [1,868-2,917]	**<0.001**
**CRP, mg/dL**	84 [24.3-140.7]	3 [1.1-7.6]	**<0.001**

Abbreviations: CRP: C-reactive protein**;** HDL-c: high-density cholesterol; LDL-c: low-density cholesterol; NA: not applicable TC: total cholesterol; TC/HDL-c: total cholesterol/high density cholesterol ratio; TG: triglycerides

Values are reported as median (IQR). Significant values ($p < 0.05$) are **bold**

[a] Lipid profiles from 1st day of hospital admission

[b] Median follow-up time of the global COVID-19 cohort was 79 [68-93] days

[c] Median follow-up time of the global COVID-19 cohort was 514 [427-617] days

[d] Wilcoxon signed rank for paired comparisons baseline and follow-up in COVID-19 survivors

SUPPLMENTARY FIGURES

Figure S1. Mortality risk in patients with COVID-19 according to LDL-c (**A**) and LPO (**B**) levels.

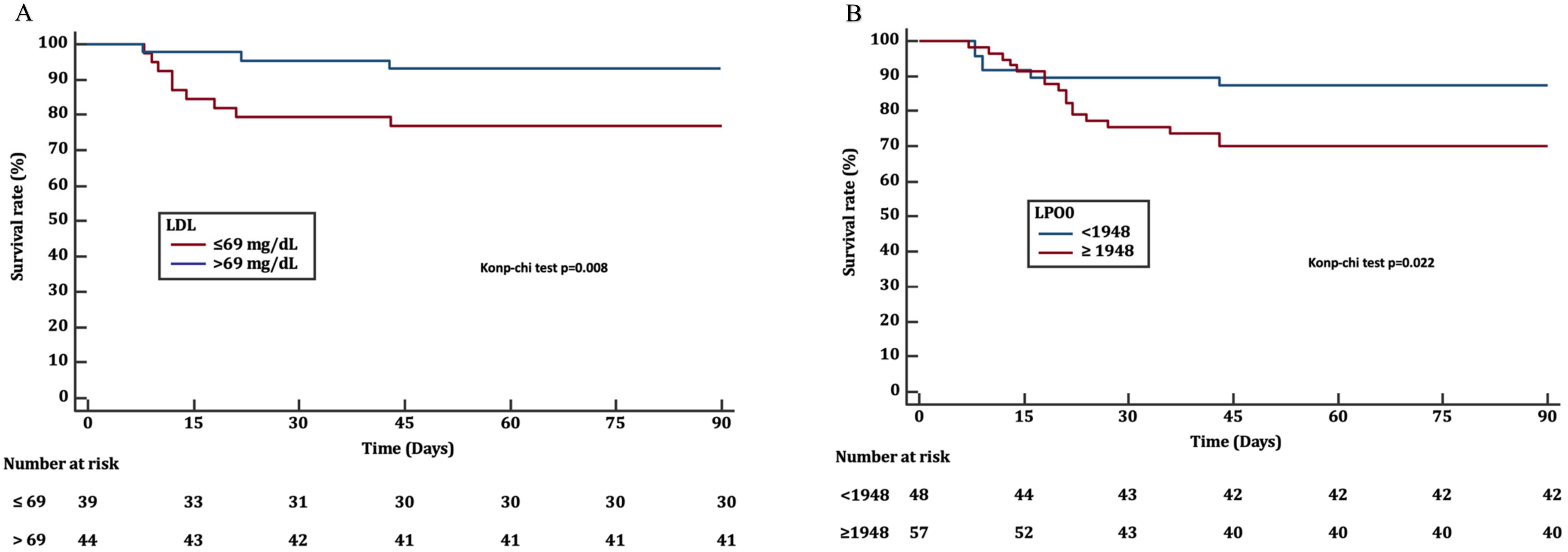

Low LDL (**A**; **red line**) and high LPO levels (**B**; **red line**) were associated with a greater 90-day all-cause mortality compared to their counterparts (**blue line**). The number at risk over time in each group is indicated. **Abbreviations:** LDL-c: low-density cholesterol; LPO: lipid peroxidation
